# Xanthogranulomatous pyelonephritis (XGPN) mimicking a “renal cell carcinoma with renal vein thrombus and paracaval lymphadenopathy”

**DOI:** 10.12688/f1000research.2-263.v1

**Published:** 2013-12-02

**Authors:** Arvind Ganpule, Jitendra Jagtap, Sanika Ganpule, Amit Bhattu, Shailesh Soni, Ravindra Sabnis, Mahesh Desai

**Affiliations:** 1 Department of Urology, Muljibhai Patel Urological Hospital, Nadiad, 387001, India; 2 Department of Radiology, Muljibhai Patel Urological Hospital, Nadiad, 387001, India; 3 Department of Pathology, Muljibhai Patel Urological Hospital, Nadiad, 387001, India

## Abstract

We present a case of Xanthogranulomatous pyelonephritis mimicking as a renal cell carcinoma. This was an elderly lady who presented with pyonephrosis due to urolithiasis. On evaluation she was found to have a space occupying mass in the right kidney. Further investigations revealed an enhancing tumor with renal vein thrombus and paracaval lymphadenopathy. Subsequent histopathology showed evidence of XGPN with no malignancy. This case report highlights the fact there are a number of imaging and clinical overlaps in the diagnosis, assessment and management of this entity.

## Case presentation

A 67 year old Hindu female presented to us in May 2010 with history of right flank pain, fever and vomiting. She had raised total leukocyte count: 16600/μL and deranged renal function (serum creatinine: 3.1mg/dL). A non-contrast CT (NCCT) scan revealed moderate hydronephrosis, right upper ureteric calculus and a well circumscribed lesion on the medial aspect of the kidney. A percutaneous nephrostomy was performed on account of the deranged renal function. Subsequently, the patient underwent a percutaneous nephrolithotomy (PCNL).

**Figure 1.**
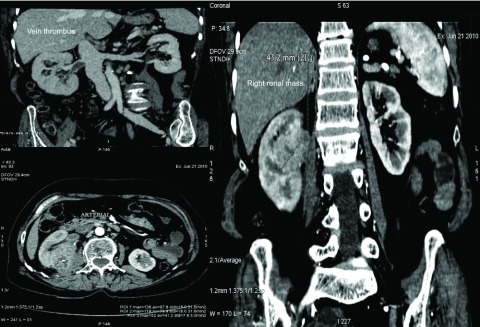
Well defined soft tissue density mass of right kidney measuring 49 × 35 × 43 mm enhancing from 33 HU to 118 HU with non-enhancing areas of necrosis.

**Figure 2.**
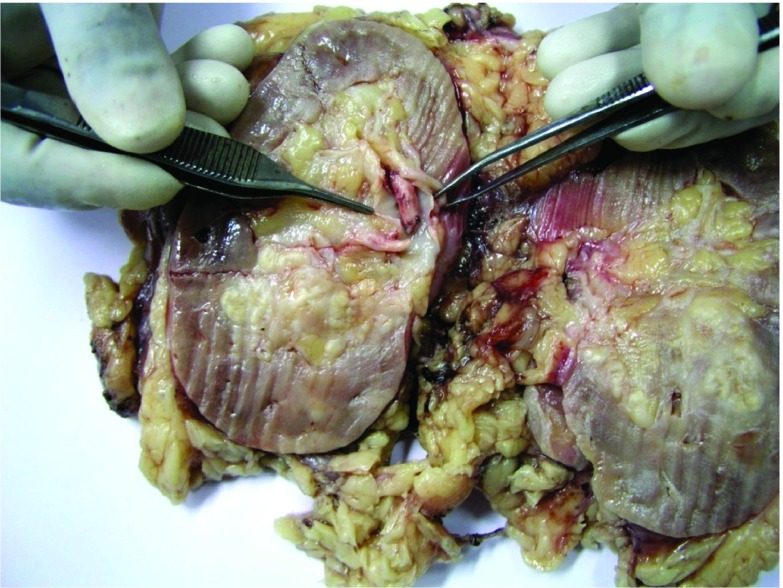
Gross specimen showing thrombus in renal vein.

**Figure 3.**
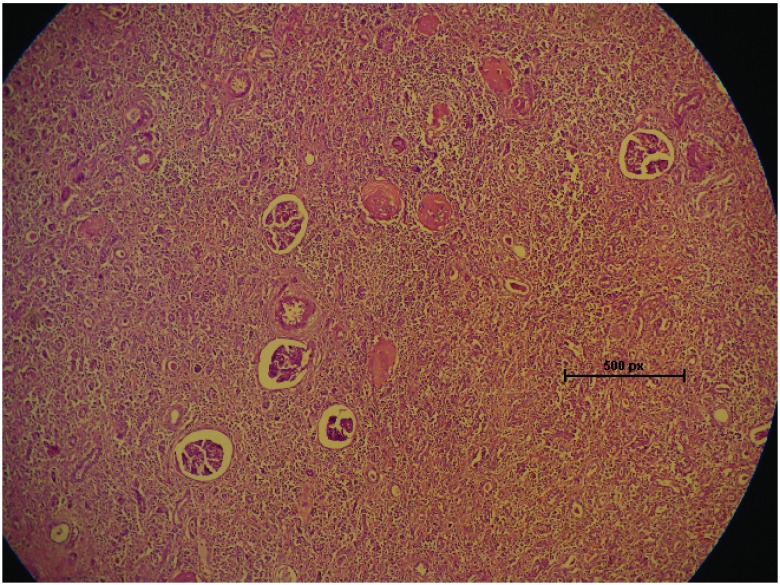
Microscopic examination at 100X magnification showing collection of foamy macrophages and inflammatory infiltrate diffusely infiltrating the renal parenchyma.

At one month from presentation and after the serum creatinine improved to 1.47mg/dL, a contrast CT revealed an enhancing mass (enhancement from 33 to 118 Hounsfield units) on the medial aspect of the kidney ( Figure 1; a contrast CT not done at initial presentation due to deranged renal function) with evidence of renal vein thrombosis and multiple paracaval lymph nodes. A provisional diagnosis of renal cell carcinoma with renal vein thrombus was made. The clinical stage was T3aN2M0. A laparoscopic radical nephrectomy was done. The gross specimen revealed evidence of renal vein thrombus and Xanthogranulomatous pyelonephritis (XGPN) ( Figure 2). On H & E (Hematoxylin & Eosin) microscopic examination, it was composed of foamy macrophages admixed with inflammatory infiltrate ( Figure 3). There was no evidence of malignancy. The patient recovered well and was discharged in stable condition after 4 days with a serum creatinine of 1.16mg/dL.

## Discussion

XGPN is an uncommon, severe, chronic suppurative renal parenchymal infection characteristically leading to renal destruction. The majority of cases are unilateral and result in a nonfunctioning, massively enlarged kidney associated with obstructive uropathy secondary to urolithiasis. XGPN has been described as a great imitator or a masquerading tumor in adults and pediatric age groups ^1, 2^. The etiological factor in this case was the renal calculus with chronic infection. The imaging findings in this case showed a significantly enhancing mass, lymph nodes and a renal vein thrombus. The mass was seen closely abutting the psoas as well. The CT findings mimicked a case of T3N2Mx renal cell carcinoma. Localised XGPN is amenable to partial nephrectomy if diagnosed preoperatively. XGPN has been found to be associated with renal cell carcinoma, papillary transitional cell carcinoma and squamous cell carcinoma and hence nephrectomy should be performed when malignancy cannot be excluded. This case highlights the need to keep XGPN as a differential diagnosis of a renal mass especially in presence of urolithiasis.

## Consent

Written informed consent for publication of clinical details and clinical images was obtained from the patient.

## References

[1] Zorzos I Moutzouris V Petraki C: Xanthogranulomatous pyelonephritis--the "great imitator" justifies its name. *Scand J Urol Nephrol.* 2002;36(1):74–6 10.1080/003655902317259418 12002363

[2] Gerber WL Catalona WJ Fair WR: Xanthogranulomatous pyelonephritis masquerading as occult malignancy. *Urology.* 1978;11(5):466–71 10.1016/0090-4295(78)90158-9 354158

