# Supplementary material for: Xanthogranulomatous pyelonephritis (XGPN) mimicking a “renal cell carcinoma with renal vein thrombus and paracaval lymphadenopathy”
Source: F1000Res. 2013 Dec 2;2:263. [Version 1] doi: 10.12688/f1000research.2-263.v1 (PMC4193392; doi:10.12688/f1000research.2-263.v1)
Supplement: Peer review history

# Peer review: Referee response for version 1

**DOI:** 10.5256/f1000research.3019.r6021

**Published:** 2014-09-04

**Authors:** Daron Smith

**Affiliations:** 1 Institute of Urology, University College London Hospitals NHS Trust, London, UK

The authors present a case of XPN (Xanthogranulomatous Pyelonephritis) that was believed to be a renal cell carcinoma based on imaging. The propensity for XPN to "imitate" renal malignancy is well established. Indeed, I wrote a case report many years ago when, in addition to the imaging apparently suggesting a renal cell carcinoma, there was an incidental small RCC in the same kidney! ( Smith RD *et al*., 2000) It would be worth emphasizing what is unique/important about this case, and the learning message that follows. Is it the apparent vascular invasion with thrombus in the vein that the authors wish to highlight?

I have a few other suggestions. In the abstract, "tumour" should be changed to "mass". The patient did not have a renal tumour in the sense of a cancer as this word is often used. “Tumour”, in its most frequently used sense of malignancy, may cause confusion to anyone reading the abstract, believing this was an renal cancer with tumour thrombus.

I would prefer to see the creatinine expressed in SI units (umol/L) and an eGFR given as well as the units in mg/dL.

I have read this submission. I believe that I have an appropriate level of expertise to confirm that it is of an acceptable scientific standard, however I have significant reservations, as outlined above.

# Peer review: Referee response for version 1

**DOI:** 10.5256/f1000research.3019.r3601

**Published:** 2014-02-25

**Authors:** Stefanos Kachrilas

**Affiliations:** 1 Urology Department, Bart's Health NHS Trust, London, UK

- The topic of this case review is not especially novel, and the information provided is unlikely to be useful to other practitioners, as the clinical entity of XGPN is well documented in the existing literature.
- The background, history, presentation, physical examination, and diagnostic tests are appropriately presented.
- The authors have not commented on the post-operative management of the patient.

I have read this submission. I believe that I have an appropriate level of expertise to confirm that it is of an acceptable scientific standard.

# Peer review: Referee response for version 1

**DOI:** 10.5256/f1000research.3019.r3093

**Published:** 2014-01-15

**Authors:** M Hammad Ather

**Affiliations:** 1 Section of Urology, Aga khan University, Karachi, Pakistan

The authors present an uncommon clinical situation where XGPN mimicked a renal tumor. XGPN is indeed a rare type of renal infection characterised by granulomatous inflammation with giant cells and foamy histiocytes. It has been shown in many case reports and small case series to mimic an infiltrative malignancy. Renal vein thrombus has also been described in many previous reports.

I have few other observations that the authors may like to address. What were the findings on nephroscopy, and did the surgeon take any biopsies of the suspicious lesion? Did you noticed any xanthoma cells in the urine? The lady has a classical presentation of an XGPN (gender, obstructing middle age ... was she diabetic or immunocompromised in any way?) stone until the contrast study. Did the investigators consider a biopsy prior to planning a nephrectomy for a fair functioning kidney?

I have read this submission. I believe that I have an appropriate level of expertise to confirm that it is of an acceptable scientific standard, however I have significant reservations, as outlined above.

# Response

**Published:** 2014-01-20

**Authors:** Jitendra Jagtap

**Affiliations:** Muljibhai Patel Urological Hospital, India

Thank you for your review Dr. M H Ather. Please find below the response to the comments:

- On nephroscopy during PCNL there were no suspicious lesions noted within the pelvicalyceal system so the surgeon did not take any biopsies. Absence of suspicious findings was double checked by reviewing the intraoperative video of this patient.
- Urine examination did not reveal the presence of any xanthoma cells.
- No, the lady was neither diabetic or immunocompromised.
- Biopsy was not considered, as it would not have altered the further management as the mass had features suggestive of malignancy on radiology namely: significant enhancement from 33 to 118 Hounsfield units, presence of renal vein thrombus, and multiple enlarged lymph nodes obviating the possibility of a nephron sparing procedure. The current roles of renal biopsy as outlined in various guidelines include: confirmation of diagnosis of radiologically indeterminate renal masses; obtaining histology of incidentally detected renal masses in patients who are candidates for nonsurgical treatment (active surveillance, ablative therapies); and selection of the most appropriate targeted therapy for metastatic renal tumours depending upon the histology ( Ljungberg B *et al.*, 2013; Novick A *et al*., 2010; Herts BR & Baker ME, 1995; Campbell SC *et al*., 1997; Volpe A *et al*., 2007). Also XGPN has been found to be associated with renal cell carcinoma, papillary transitional cell carcinoma and squamous cell carcinoma, and hence nephrectomy should be performed when malignancy cannot be excluded ( Tolia BM *et al*., 1981; Schoborg TW *et al*., 1980).
